# Improving the Quality of Health Care Services for Adolescents, Globally: A Standards-Driven Approach

**DOI:** 10.1016/j.jadohealth.2015.05.011

**Published:** 2015-09

**Authors:** Manisha Nair, Valentina Baltag, Krishna Bose, Cynthia Boschi-Pinto, Thierry Lambrechts, Matthews Mathai

**Affiliations:** a National Perinatal Epidemiology Unit, Nuffield Department of Population Health, University of Oxford, Oxford, United Kingdom; b Department of Maternal, Newborn, Child and Adolescent Health, World Health Organization, Geneva, Switzerland

**Keywords:** Adolescents, Health care, Quality of care, Global standards

## Abstract

**Purpose:**

The World Health Organization (WHO) undertook an extensive and elaborate process to develop eight Global Standards to improve quality of health care services for adolescents. The objectives of this article are to present the Global Standards and their method of development.

**Methods:**

The Global Standards were developed through a four-stage process: (1) conducting needs assessment; (2) developing the Global Standards and their criteria; (3) expert consultations; and (4) assessing their usability. Needs assessment involved conducting a meta-review of systematic reviews and two online global surveys in 2013, one with primary health care providers and another with adolescents. The Global Standards were developed based on the needs assessment in conjunction with analysis of 26 national standards from 25 countries. The final document was reviewed by experts from the World Health Organization regional and country offices, governments, academia, nongovernmental organizations, and development partners. The standards were subsequently tested in Benin and in a regional expert consultation of Latin America and Caribbean countries for their usability.

**Results:**

The process resulted in the development of eight Global Standards and 79 criteria for measuring them: (1) adolescents' health literacy; (2) community support; (3) appropriate package of services; (4) providers' competencies; (5) facility characteristics; (6) equity and nondiscrimination; (7) data and quality improvement; and (8) adolescents' participation.

**Conclusions:**

The eight standards are intended to act as benchmarks against which quality of health care provided to adolescents could be compared. Health care services can use the standards as part of their internal quality assurance mechanisms or as part of an external accreditation process.

**Implications and Contribution**

This article presents the eight Global Standards developed by the World Health Organization to improve quality of health care services for adolescents and a description of the extensive and elaborate process used to develop the standards. The standards will be implemented across countries, globally, as benchmarks for quality of health care provided to adolescents.

Presently, there are 1.2 billion adolescents globally [1] who are a valuable resource for countries [2], but they are also at an increased risk of mortality and morbidity due to intentional and unintentional injuries, mental health problems, pregnancy-related complications and various life-threatening communicable diseases (such as Human Immunodeficiency Virus (HIV), Hepatitis B, etc.) [3,4]. Although decreasing high-risk behavior, preventing mortality and morbidity, and improving health among adolescents require a life-course action [2], the role of health systems in providing adequate quality of care is undeniable. Health systems globally have to be responsive to the unique demands of young people and focus on improving quality alongside coverage of youth-friendly health care services [5] such that these services are acceptable, effective, efficient, equitable, and safe for adolescents [6].

Evidence from both high- and low-income countries shows that adolescents and young adults face many barriers which prevent their use of health services [7–13]. Pockets of excellent practice exist, but, overall, services need significant improvement [14]. The World Health Organization's (WHO) report, “Health for the world's adolescents: a second chance in the second decade,” suggests that to make progress toward universal health coverage, ministries of health and the health sector more generally will need to transform how health systems respond to the health needs of adolescents. It recommends developing and implementing national quality standards and monitoring systems as one of the actions necessary to make this transformation [14]. The WHO undertook an extensive and elaborate process through collaboration with many departments within the organization, other partner organizations and stakeholders from several countries globally, to develop eight Global Standards to improve quality of health care services for adolescents. The objectives of this article are to present the Global Standards and their method of development.

## Methods used for developing the Global Standards

A standard is a statement of a defined level of quality in the delivery of services that is required to meet the needs of intended beneficiaries [15,16], in this context safe, accessible, acceptable, appropriate equitable, effective, and efficient health care [6]. Standardization in general is a way to minimize variability and ensure a minimal required level of quality to protect users and is used in many sectors and various aspects of health care. In health care, a standards-driven approach has been used to allow health services to realize aspirational but achievable goals through assisting in the implementation of appropriate practices and guiding continuous quality improvement [16,17]. The Global Standards were developed through a four-stage process which included the following: (1) conducting needs assessment; (2) developing the Global Standards and their criteria; (3) expert consultations; and (4) assessing the usability of the Global Standards. The aim of these global standards was to improve overall quality of health care for adolescents irrespective of the conditions for which health care may be sought. The standards and criteria do not focus on any particular specialty of health care (such as reproductive health, mental health, communicable and noncommunicable diseases, injuries, etc.), whereas condition-specific aspects are dealt with through the promotion of effective care including the use of clinical practice guidelines and protocols (Standard no. 4). Thus, these are primarily service standards which can be adopted to improve the quality of general and specialist care at all levels (primary, secondary and tertiary).

### Needs assessment

The aspect of quality in health care services for adolescents is not well understood. The challenge is that, in general there is no single definition or framework for improving quality of health care. Although there is existing knowledge about the factors that could promote or hinder quality improvements in health care services, the evidence is scattered in a large number of published and unpublished literature. Therefore, as a first step, a meta-review of high-quality systematic reviews was conducted to examine the facilitators and barriers to improving quality of health care for adolescents. Both published and unpublished systematic reviews and/or meta-analyses of interventions for adolescents that focused on improving quality of health care from January 2000 to June 2013 were included. Adolescents were defined as people in the age group of 10–19 years as recommended by the WHO [18]. Considering that there is no single definition of optimal quality of health care, the six dimensions of desired health care performance suggested by the Institute of Medicine—effective, efficient, accessible, acceptable/patient-centered, equitable, and safe [6] were used as surrogate markers of “quality of health care.” No language restriction was applied. Systematic reviews/meta-analyses of therapies and drug interventions and of specific disease or health conditions were excluded. Other exclusion criteria were systematic reviews withdrawn by the journal/authors due to any reason, systematic reviews of specific diseases, and systematic reviews of health promotion strategies/programs undertaken by sectors other than the health sector. Details of database searches and key words are provided in Appendix-A (supplementary file).

Standard data extraction formats were used to collect information on methods, participants, intervention, and outcome. The 11-item assessment of multiple systematic reviews tool was used to assess the methodological quality of the systematic reviews included [19]. Data analysis was conducted through qualitative synthesis of the extracted data. Considering the heterogeneity of outcomes, interventions, and settings, no attempt was made to conduct a meta-regression of the meta-analyses data. The method used in the meta-review was similar to that used to identify the facilitators and barriers to improving quality of care for mothers, newborns, and children published elsewhere [20].

In addition to the meta-review of literature, two online global surveys were conducted by the WHO in 2013, one with primary health care providers and another with adolescents. The first survey was open to all primary health care providers across the world from 15 July to 7 October to collect input on the facilitators and barriers to improving the quality of health care services for adolescents. This survey was part of the process to develop the global report on health of the world's adolescents [14] and included questions on accessibility; quality improvement; providers' skills; facility policies regarding equity, confidentiality, privacy and informed consent, financial protection, and users' fees; and other aspects relevant to quality of care for adolescents.

The second online survey undertaken as part of the process of developing the global adolescent health report [14] was open to everyone in the age group of 12–19 years. The objective of this survey was to obtain adolescents' perspectives on the health issues affecting them, and it was conducted via an open access online survey in the six official United Nations languages. The key areas focused were as follows: (1) adolescents' understanding of health, including the factors that influence health; (2) adolescents' views about priorities among health issues; (3) barriers to the use of health services; and (4) adolescents' opinions about how their health could be improved.

### Developing the Global Standards

The Global Standards were developed based on the needs assessment informed by the meta-review and online surveys of primary health care providers and adolescents in conjunction with analysis of 26 national standards for providing health care services to adolescents from 25 countries: Bangladesh, Bhutan, Burkina Faso, Congo, Ethiopia, Ghana, India, Indonesia, Kyrgyzstan, Lesotho, Malawi, Mongolia, Myanmar, Nicaragua, Philippines, Moldova, South Africa, Sri Lanka, Tajikistan, Tanzania, Thailand, United Kingdom (England, Scotland), Ukraine, Vietnam, and Zambia. The analysis identified the most common standards (a standard was considered common if it was found in at least 50% of reviewed countries' standards) and their criteria (a criterion was considered common if it was found in at least 25% of reviewed countries' criteria). A criterion was defined as a measurable element of a standard that defines a characteristic of the service (input criterion) that needs to be in place or implemented (process criterion) to achieve the defined standard (output criterion). A WHO interdepartmental technical working group that included representatives from the Department of Maternal, Newborn, Child and Adolescent Health, the Department of Reproductive Health and Research, and the Department of Immunization, Vaccines and Biologicals, reviewed the initial draft of the Global Standards.

### Peer review

The final document was reviewed by the WHO regional and country offices (WHO Country Office in Ukraine, WHO Regional Office for Africa, WHO Regional Office for Europe, WHO Regional Office for the Western Pacific); 20 external reviewers representing national and international experts from governments and academia from Australia, Estonia, India, Moldova, and Tanzania; and nongovernmental organizations and development partners (including Evidence To Action, International Planned Parenthood Federation, Jhpiego, LoveLife, Pathfinder International, Save the Children, United Nations Children's Fund, United Nations Population Fund). Peer reviewers were invited to comment on any aspect of the document and provide suggestions for improvement, as well as provide specific comments on four key questions included in Box-1.

### Assessing the usability of the Global Standards

The usability of the standards was tested during two regional expert consultations on the development of Adolescent Sexual and Reproductive Health (ASRH) Standards in Latin America in November 2014, and in the Caribbean in April 2015. The consultations included directors of adolescent and youth programs from ministries of health and development organizations from Antigua and Barbuda, Barbados, Belize, Brazil, Chile, Colombia, Costa Rica, Cuba, Dominican Republic, Ecuador, El Salvador, Guatemala, Grenada, Guyana, Honduras, Jamaica, Mexico, Nicaragua, Panama, Paraguay, Peru, St. Lucia, St. Vincent, Suriname, Trinidad and Tobago, Uruguay, and Venezuela. The WHO Global Standards were used as a basis for adaptation to develop regional ASRH standards for Latin American and Caribbean countries. Participants worked in groups to answer the following questions: (1) Are the standard statements adequate for regional ASRH standards, and if not what changes are proposed? and (2) Are the criteria within each standard adequate for regional ASRH standards, and if not what changes are proposed?

A third field test was conducted in Benin where national stakeholders used the global document, in conjunction with a situation analysis, to develop national standards.

## Results

### Needs assessment—meta-review

**Figure 1:**
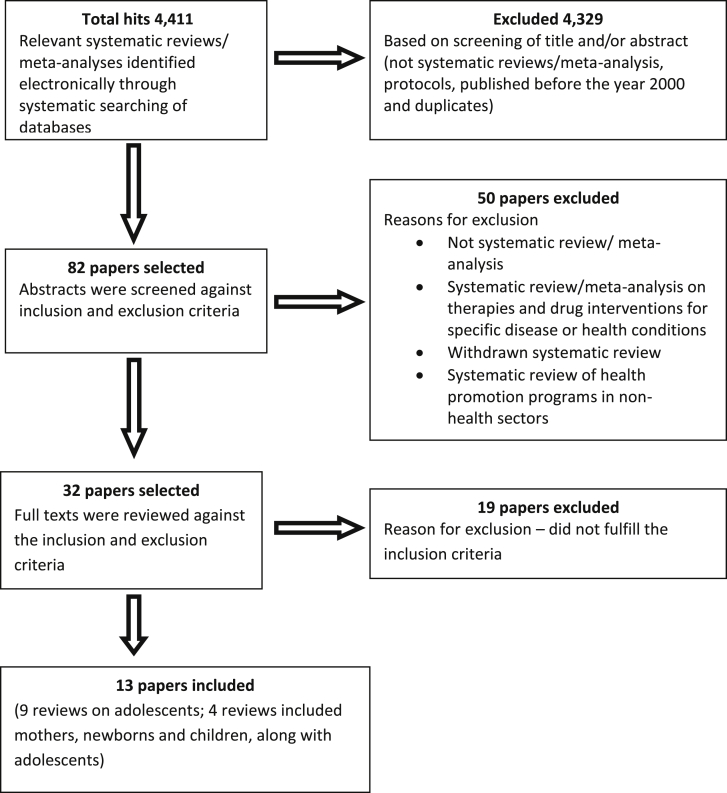
Schematic presentation of the process of selection of published literature for the meta-review.

A systematic searching of the databases of published literature gave a total of 4,411 hits; the abstracts were screened against the inclusion and exclusion criteria; and finally, 13 full texts were included (details of the search is provided in Figure 1). No unpublished systematic reviews/meta-analyses were found that fulfilled our selection criteria. The list of included and excluded studies (with reasons for exclusion) is provided in Appendix-B (supplementary file). The citations were managed using EndNote X5 (StataCorp, College Station, TX).

The 13 systematic reviews included a total of 245 studies with a range of study designs and sample sizes from high-, middle-, and low-income countries (Table 1). The systematic reviews included 13 interventions that focused on one or more of the six dimensions of desired health care performance (effective, efficient, accessible, acceptable, equitable, and safe). The assessment of multiple systematic reviews scores ranged from three to 11 with no systematic review that scored below three (used as a cutoff for adequate quality of systematic reviews). The included systematic reviews mainly focused on health promotion, adherence to treatment, prevention of teenage pregnancy, and support for teenage mothers. Although WHO defines adolescents as people in the age group of 10–19 years, the systematic reviews included adolescents and young people in the age group of 10–24 years.

Analysis of the reviews showed that there are a number of existing facilitators and barriers to improving quality of health care for adolescents related to provision of information, communication with providers, engagement with health care services, regulations and standards, organizational capacity, and satisfaction. The barriers were related to both access and utilization of care. Equity in access to care by adolescents belonging to minority communities was found to be a specific issue [21], but there is a general requirement for health care services to be more accessible for adolescents. The main facilitator to improving access was to make the services available at schools and communities [21–23].

The challenges for utilization of health care services were related to the process of delivery and included judgmental, uncaring, and disrespectful attitude of providers which shaped the perceptions of adolescents about the quality of care and increased their distrust on health care provision [21,22]. It was observed that confidentiality in care provision, trust in providers, and comfort and support from providers [21,24] were key, and facilitators were mainly the interventions that focused on improving these aspects of care. Thus, the facilitators were improved user–provider interpersonal communication [23,25], continuous communication with providers [26], role models [23], cultural sensitivity [21,22], and youth-friendly health care services [5,27]. Receiving adequate information was considered important by adolescents, and any effort in this area including text messages, information support from health care providers, and through the mass-media was observed to improve acceptability of health care services among adolescents [23,27–30]. However, one key barrier to effective communication and providing information was language, especially among minority population groups [21].

### Needs assessment—Online survey of primary health care providers and adolescents

The health providers' online survey was answered by 735 respondents from 81 countries representing all six WHO regions. Data from the survey confirmed that the findings of the meta-review adequately reflected areas that in respondents' opinion needed improvement. In addition, the health care providers raised concerns about their ability to dedicate sufficient time to work effectively with their adolescent clients and the use of evidence-based protocols. The commonly available guidelines and protocols were related to providing information and counseling on contraception, including emergency contraception (73%), vaccinations other than Human Papillomavirus (72%), and information and counseling on nutrition (71%). About half of the respondents indicated the availability of protocols for preconception care (54%), care and support of adolescents who have been physically or sexually assaulted (54%), and screening and counseling for mental health problems (58%), chronic illness (58%) and common endemic diseases (55%). Less than half reported the availability of guidelines and protocols on acne, dental care, abortion services and treatment, and care and support for HIV-positive adolescents. However, because of the nature of the survey question which enquired only about availability, we cannot infer whether the available guidelines were being used. Overall, 69% of providers answered that they need more guideline/protocols to support them in providing services to adolescents, and the four priority areas mentioned were mental health (34%), substance misuse (28%), sexual/reproductive health (28%), and domestic/school violence (26%).

A total of 1,143 adolescents from 104 countries participated in the adolescents' consultation. The highest number of participants was from the United States of America followed by Bangladesh, United Kingdom, Indonesia, Australia, India, Canada, Mexico, Morocco, France, Malawi, and Malaysia. Almost half of the respondents (49%) were from low- and middle-income countries; most were aged ≥15 years and attended school; and 63% were females. The following five main themes emerged from the consultations:

i. Adolescents understand the importance of health, are conscious of the main health issues affecting them and should therefore be engaged in addressing their health care needs.
ii. There is an increased demand for information about health and health care among adolescents.
iii. The adolescents often reported families to be the most influential source of health information and a crucial determinant of their well-being.
iv. Although sexual and reproductive health services were considered important, adolescents demanded other services, in particular, those addressing their mental health needs.
v. Proximity to health care services and their costs and quality influenced adolescents' use of health care services.

### Developing the Global Standards

Analysis of 26 national standards from 25 countries showed that the most common standards (defined as found in at least 50% of reviewed countries' standards) were related to aspects of service provision, workforce capacity, community involvement, availability of drugs, supplies and technologies, and youth participation. Most frequently, national standards stated the necessity of a comprehensive package of services (all 26 national standards), effective care (25 standards), acceptable care (24 standards), and accessible care (23 standards), but equity aspects were mentioned in only nine countries' standards.

Lessons learned from the analysis were reviewed in conjunction with the facilitators and barriers identified through the meta-review and the needs defined by the health care providers from 81 countries and adolescents from 104 countries to develop eight Global Standards for improving quality of health care services for adolescents. Initially, the areas that emerged to be the most important from the analysis of the national standards were proposed as “core” standards; and others such as equity, financial protection, and youth participation were proposed as “additional” standards. However, the WHO interdepartmental technical working group decided that having “core” and “additional” standards might be confusing for countries and the fact that some aspects (e.g., youth participation or equity) were not frequently mentioned meant rather low awareness of their importance among policy makers and health care planners than their importance per se. For example, financial barriers and engaging in planning for their own health needs were important themes that emerged from the online adolescent survey.

### Key inputs from reviewers

No radical changes to the standards were recommended during the peer review, but there were useful and consistent suggestions provided for better emphasis on a human rights–based approach, positioning of standards' criteria to avoid redundancies and ensure better content validity, and structure of the document which added considerably to the clarity of the document.

### Global Standards for improving the quality of health care services for adolescents

The eight Global Standards are described in the following, and the criteria for measuring them are defined in Table 2. The criteria are divided into “input” (characteristics of the health services such as setting, material and human resource, organizational structures, regulations and standards), “process” (quality of implementation of the services), and “output” (achieving the defined standard). The expected outputs from these standards include improvements in “input” and “process” of health care for adolescents along with improvements of clinical and behavioral outcomes. Therefore, the outputs for health care delivery, in particular, were restatements of criteria included under “process” for a number of standards.

#### Standard 1: Adolescents' health literacy

The health facility implements systems to ensure that adolescents are knowledgeable about their own health, and they know where and when to obtain health services.

#### Standard 2: Community support

The health facility implements systems to ensure that parents, guardians, and other community members and community organizations recognize the value of providing health services to adolescents, and support such provision and the utilization of services by adolescents.

#### Standard 3: Appropriate package of services

The health facility provides a package of information, counseling, diagnostic, treatment, and care services that fulfill the needs of all adolescents. Services are provided in the facility and through referral linkages and outreach.

#### Standard 4: Providers' competencies

Health care providers demonstrate the technical competence required to provide effective health services to adolescents. Both health care providers and support staff respect, protect, and fulfill adolescents' rights to information, privacy, confidentiality, nondiscrimination, nonjudgmental attitude, and respect.

#### Standard 5: Facility characteristics

The health facility has convenient operating hours, a welcoming and clean environment, and maintains privacy and confidentiality. It has the equipment, medicines, supplies, and technology needed to ensure effective service provision to adolescents.

#### Standard 6: Equity and nondiscrimination

The health facility provides quality services to all adolescents irrespective of their ability to pay, age, gender, marital status, education level, ethnic origin, sexual orientation, or other characteristics.

#### Standard 7: Data and quality improvement

The health facility collects, analyses, and uses data on service utilization and quality of care, disaggregated by age and gender to support quality improvement. Health facility staff is supported to participate in continuous quality improvement.

#### Standard 8: Adolescents' participation

Adolescents are involved in the planning, monitoring, and evaluation of health services and in decisions regarding their own care, as well as in certain appropriate aspects of service provision.

### Usability of the Global Standards

Field testing not only showed the adequacy of standards and their criteria to the Latin American and Caribbean countries' context, but also showed that as a tool, the Global Standards are easily adaptable in a relatively short timeframe and can thus be good guide for consultation with broader groups of stakeholders. The changes proposed by participants to the Global Standards pertained mainly to the improvement of Spanish translation of Global Standards. Based on the Global Standards, the participants suggested that the regional standards should include a comprehensive package of services and not simply limit it to adolescents' sexual and reproductive health. They also felt that the regional standards lacked explicit mention of adolescents' knowledge of their rights in health care and was therefore updated based on criterion 34 of the Global Standard-4 (Table 2) to address this omission.

In the past, development of national standards required external expertise, whereas in Benin, the national team completed the process without external help. In the current draft, Benin standards and criteria are similar to the global ones, and the implementation plan reflects key actions, although in a less detailed manner than are outlined in the global guidance.

## Discussion

The extensive process undertaken resulted in the development of eight Global Standards and 79 criteria for measuring the characteristics of the services and quality of implementation to achieve the desired standards. The standards were as follows: (1) adolescents' health literacy; (2) community support; (3) appropriate package of services; (4) providers' competencies; (5) facility characteristics; (6) equity and nondiscrimination; (7) data and quality improvement; and (8) adolescents' participation. These standards are intended to act as benchmarks against which quality of health care provided to adolescents could be compared, and the criteria developed could help to make the comparisons. Health service providers can use the standards as part of their internal quality assurance mechanisms or as part of an external accreditation process [15,31].

Many countries to date have moved toward a standards-driven approach to improve the quality of care for adolescents [5,32–36]. Standards are developed to be applied across the broad range of services such as primary care, general practice and community-based services, government and nongovernment services, and those in the private sector [15–17,31,37]. However, it is recognized that quality improvement is a continuous process and that the standards would be a “living document” that will further evolve as services progressively strive to meet relevant and expected standards of care [17].

Service standards intend to guide individual facility on how to improve its quality of care for adolescent clients and foster national- and district-level actions to support the facility in doing so. Being developed nationally, or sometime regionally, they may require adaptations to capture the peculiarity of a single clinic/practice setting or large hospitals and health care systems. Furthermore, the standards and the criteria proposed relate mainly to the traditional systems of health care delivery and may need some modification or adaptation to align with electronic health care delivery which is being tested and increasingly used in countries. However, the primary objectives are likely to remain unchanged.

It is recognized that developing and implementing national quality standards and monitoring systems is just one part of the transformation that health systems need to undergo to better respond to adolescent health and development needs [14]. Improving the quality of care at primary and referral level facilities cannot succeed without strengthening all pillars of the health system: governance, financing, strengthening workforce capacity, and ensuring that the necessary drugs, supplies and technology are available. Therefore, apart from actions in the facility and community, national- and district-level actions will be necessary in each of the health system pillars to enable health care providers and managers to implement the standards and their criteria.

### Strengths and limitations of the processes

The major strength of the process of developing the “Global Standards for improving the quality of health care for adolescents is the use of a bottom-up approach through extensive consultations of published and unpublished literature; existing national standards in 25 countries; health service providers from 81 countries; experts from the academia, governmental, nongovernmental, and development partners; and with adolescents from 104 countries across the globe. Although the online surveys with health care providers and adolescents are prone to self-selection bias and neither were representative of the target population, they provided a valuable means to engage with providers and adolescents in defining their health care needs. The agreement of the themes generated through these online surveys with that of the findings of the meta-review increased reliability of the findings of the needs assessment.

However, it is acknowledged that health service standards represent only one component of different quality, safety, and performance frameworks for health systems [15,17] and services might be at different stages, such that some standards will be routine practice for some and aspirational for others [15,17]. However, standards are designed to be assessed [15–17,31,37,38], and it is expected that the eight standards proposed will further develop with the process of implementation and will be adapted by countries at national and regional levels to suit their specific needs.

## Figures and Tables

**Table 1:** Description of the studies included in the meta-review

Sl. No.	Citation	Systematic review or grey literature	Description of the studies included in the reviews						AMSTAR score
			Number of studies included	Type of studies included	Total sample (where available)	Country category	Names of countries (where available)	Rural/urban	
1	Beach et al., 2006 [30]	Systematic review	27	RCTs	NR	High income	United States of America	NR	4
2	Elster et al., 2003 [21]	Systematic review	29	Cross-sectional surveys (national) and longitudinal studies	579–158,025	High income	United States of America	NR	4
3	Militello et al., 2012 [28]	Systematic review	8	RCT and one quasi-experimental design	36–126	High income	United Kingdom, United States of America, New Zealand, and Austria	NR	7
4	Oringanje et al., 2009 [39]	Systematic review	41	RCTs	95, 662	All groups	Nigeria, United States of America, England, Canada, Italy, Mexico, and Scotland	NR	11
5	Salema et al., 2011 [23]	Systematic review	20	RCTs	NR	High income	United States of America, Canada, United Kingdom, and Netherlands	NR	5
6	Mason-Jones et al., 2012 [22]	Systematic review	27	Controlled before-and-after, cross-sectional, cohort	104–6,080	High income	United Kingdom, United States of America, and Canada	NR	4
7	Speizer et al., 2003 [27]	Not reported as a systematic review, but methods used comply with that of systematic review methods	41	RCTs and quasi-experimental	84–4,777	All groups	Saudi Arabia, Brazil, Philippines, Peru, Nigeria, Jamaica, South Africa, Uganda, Tanzania, Zimbabwe, Chile, Mexico, Namibia, Thailand, Paraguay, Botswana, Cameroon, Guinea, and India.	Both	4
8	Dean et al., 2010 [26]	Systematic review	17	RCTs and quasi-experimental	20–318	NR	NR	NR	5
9	Letourneau et al., 2004 [29]	Not reported as a systematic review, but methods used comply with that of systematic review methods	19	RCTs, post hoc evaluation and quasi-experimental	12–5400	NR	NR	NR	4
10	Stinson et al., 2009 [24]	Systematic review	9	RCTs (7), pilot RCTs (1), quasi-experimental (1)	1,415 children, adolescents, and adults (range 24–438)	High income	China, Canada, United States of America, and Germany	NR	7
11	Ruiz-Mirazo et al., 2012 [40]	Systematic review	8	Three RCTs, five cohort studies	RCTs = 1,903, cohort = 1,106	All groups	United States of America and Iran	NR	3
12	Hall Moran et al., 2007 [25]	Systematic review	3	RCT (1), qualitative study—in-depth interviews (2)	RCT = 136, qualitative (60 adolescents in one and two in the other)	High income	United Kingdom and Australia	NR	4
13	Ambresin et al., 2013 [5]	Systematic review	22	Prospective cohort (5), cross-sectional (10), qualitative (7)	Quantitative 24–8855, qualitative 14–60	All groups	United States of America, United Kingdom, New Zealand, Kenya, Zimbabwe, Mongolia, Ireland, Australia, Canada, Jordan, Switzerland	NR	9

AMSTAR = assessment of multiple systematic reviews; NR = not reported; RCT = randomized controlled trials.

**Table 2:** Criteria for the Global Standards for improving quality of adolescent health care

Input	Process	Output
Standard 1: adolescents' health literacy		

1. The health facility has a signboard that mentions operating hours.^a^ 2. The health facility has in the waiting area up-to-date information, education, and communication materials specifically developed for adolescents. 3. Health care providers have competencies^b^ to provide health education to adolescents and to communicate about health^c^ and available services (health, social and other services^d^). 4. The health facility has outreach workers^e^ who are trained to provide health education to adolescents in the community. 5. The health facility has a plan for outreach activities and/or involvement of outreach workers in activities to promote health and increase adolescents' use of services.	6. Health care providers provide age and developmentally appropriate health education and counseling to adolescent clients and inform them about the availability of health, social services, and other services. 7. Outreach activities to promote health and increase adolescents' use of services are carried out according to the health facility's plan.	8. Adolescents are knowledgeable about health. 9. Adolescents are aware of what health services are being provided, where and when they are provided, and how to obtain them.
Standard 2: community support		
10. Health care providers have competencies^b^ and support materials to communicate with parents, guardians, and other community members and organizations about the value of providing health services to adolescents. 11. The health facility has an updated list of agencies and organizations with which it partners to increase community support for adolescents' use of services. 12. The health facility has a plan for outreach activities and/or involvement of outreach workers in activities to increase gatekeepers' support for adolescents' use of services.	13. The health facility engages in partnerships with adolescents, gatekeepers, and community organizations to develop health education and behavior-oriented communication strategies and materials and plan service provision. 14. Health care providers inform parents/guardians visiting the health facility about the value of providing health services to adolescents. 15. Health care providers and/or outreach workers inform parents/guardians and teachers during school meetings about the value of providing health services to adolescents. 16. Health care providers and/or outreach workers inform youth and other community organizations about the value of providing health services to adolescents.	17. Gatekeepers and community organizations support the provision of health services to, and their utilization by, adolescents.
Standard 3: appropriate package of services		
18. Policies are in place that define the required package^f^ of health information, counseling, diagnostic, treatment and care services and enable its provision. 19. Policies and procedures^g^ are in place that identifies which health services are provided in the health facility and which in community settings such as schools.^h^ 20. Policies and procedures are in place that describes the referral system to services within and outside the health sector, including provisions for transition care for adolescents with chronic conditions.	21. Health care providers provide the required package of health information, counseling, diagnostic, treatment, and care services in the facility and/or in community settings, in line with policies and procedures.^i^ 22. Service providers refer adolescents to the appropriate service and level of care according to local policies and procedures, and follow the policies for transition care.	23. The health facility provides a package of health services that fulfills the needs of all adolescents, in the facility and/or through referral linkages and outreach.
Standard 4: providers' competencies		
24. Health care providers and support staff of the required profile^j^ are in place. 25. Health care providers have the technical competencies^k^ necessary to provide the required package of services. 26. Healthcare providers have been trained/sensitized on the importance of respecting the rights of adolescents to information, privacy, and confidentiality, respectful, nonjudgmental and nondiscriminatory health care. 27. Providers' obligation and adolescents' rights^l^ are clearly displayed in the health facility. 28. Up-to-date decision support tools (guidelines, protocols, algorithms) that cover topics of clinical care in line with the package of services are in place. 29. A system of supportive supervision is in place to improve health care providers' performance. 30. A system of continuous professional education that includes an adolescent health care component is in place to ensure lifelong learning.	31. Health care providers follow evidence-based guidelines and protocols in delivering care to adolescents. 32. Health care providers and support staff relate to adolescents in a friendly manner and respect their rights to information, privacy, and confidentiality; nondiscrimination; nonjudgmental attitude; and respectful care.	33. Adolescents receive effective^m^ health services. 34. Adolescents receive services in a friendly, supportive, respectful, nondiscriminatory, and nonjudgmental manner and know their rights to health care 35. Adolescents receive accurate, age-appropriate, and clear information to facilitate informed choice.
Standard 5: facility characteristics		
36. A policy is in place, including assigned responsibilities across health care providers and support staff, to ensure a welcoming and clean environment,^n^ minimize waiting times, and ensure convenient operating hours and flexible appointment procedures. 37. The facility has basic amenities (electricity, water, sanitation and waste disposal). 38. Policies and procedures to protect the privacy and confidentiality^o^ of adolescents are in place. Both health care providers and support staff know them and their own roles and responsibilities. 39. A system of procurement and stock management of the medicines and supplies necessary to deliver the required package of services is in place. 40. A system of procurement, inventory, maintenance, and safe use of the equipment necessary to deliver the required package of services is in place.	41. Health care providers offer consultations during hours that are convenient to adolescents in local communities, with or without an appointment. 42. Health care providers and support staff follow policies and procedures to protect the privacy and confidentiality of adolescents. 43. Medicines and supplies are in adequate quantities without shortages (stock-outs) and are equitably used. 44. The equipment necessary to provide the required package of services to adolescents is available, functioning, and equitably used.	45. The health facility has convenient operating hours, appointment procedures, and waiting times. 46. The health facility has a welcoming and clean environment. 47. Adolescents receive private and confidential health care at all times during the consultation process. 48. The facility has the equipment, medicines, supplies, and technology needed to ensure effective service provision to adolescents.
Standard 6: equity and nondiscrimination		
49. Policies and procedures are in place stating the obligation of facility staff to provide services to all adolescents irrespective of their ability to pay, age, gender, marital status, schooling, race/ethnicity, sexual orientation, or other characteristics. 50. Policies and procedures are in place for services that are free at the point of use, or affordable. 51. Health care providers and support staff are aware of the above policies and procedures and know how to implement them. 52. The policy commitment of the health facility to provide health services to all adolescents without discrimination, and take remedial actions when necessary, is displayed prominently in the health facility. 53. Health care providers know who are the vulnerable group(s) of adolescents in their community(ies).	54. Health care providers and support staff demonstrate the same friendly, nonjudgmental, and respectful attitude to all adolescents, regardless of age, gender, marital status, cultural background, ethnic origin, disability, or any other reason. 55. Health care providers provide services to all adolescents without discrimination, in line with policies and procedures. 56. The health facility involves vulnerable group(s) of adolescents in the planning, monitoring, and evaluation of health services, as well as in certain aspects of health service provision.	57. All adolescents—irrespective of their ability to pay, age, gender, marital status, education, ethnic origin, sexual orientation, or other characteristics—report similar experiences of care.^p^^q^ 58. Vulnerable group(s) of adolescents are involved in the planning, monitoring, and evaluation of health services, as well as in certain aspects of health service provision.^r^
Standard 7: data and quality improvement		
59. A system is in place to collect data on service utilization disaggregated by age, gender, and other sociodemographic characteristics as relevant.^s^ 60. Health care providers are trained to collect and analyze data to inform quality improvement initiatives. 61. Tools and mechanisms for self-monitoring of the quality of health services for adolescents are in place. 62. Mechanisms are in place to link supportive supervision to priorities for improvement as identified during the monitoring of the implementation of standards. 63. Mechanisms are in place for reward and recognition of highly performing health care providers and support staff.	64. The health facility collects data on service utilization disaggregated by age and gender and conducts regular self-assessments of quality of care.^t^ 65. Health care providers and support staff use data on service utilization and quality of care for action planning and implementation of quality improvement initiatives. 66. Health care providers and support staff receive supportive supervision in areas identified during self-assessments. 67. Good performance is recognized and rewarded.	68. Facility's reports to district include data on cause-specific utilization of services by adolescents by age and gender. 69. Facility's reports to districts on quality of care have a focus on adolescents. 70. Health facility staff feels supported by supervisors and motivated to comply with the standards.
Standard 8: adolescents' participation		
71. The governance structure of the facility includes adolescents. 72. There is a policy in place to engage adolescents in service planning, monitoring and evaluation. 73. Healthcare providers are aware of laws and regulations that govern informed consent, and the consent process is clearly defined by facility policies and procedures in line with laws and regulations.	74. The health facility carries out regular activities to identify adolescents' expectations about the service^u^ and to assess their experience of care, and it involves adolescents in the planning, monitoring and evaluation of health services. 75. Healthcare providers provide accurate and clear information on the medical condition and management/treatment options,^v^ and explicitly take into account the adolescent's decision on the preferred option and follow-up actions. 76. The health facility carries out activities to build adolescents' capacity in certain aspects of health-service provision.^w^	77. Adolescents are involved in planning, monitoring and evaluation of health services. 78. Adolescents are involved in decisions regarding their own care. 79. Adolescents are involved in certain aspects of health service provision.

a If there are special days and/or hours for adolescents, these should be clearly mentioned.

b Competencies are defined in a job description.

c This includes not only knowledge about an adolescent's own health status but also knowledge about positive health behaviors, risk and protective factors, and health determinants.

d Other services that adolescents might need may include shelters, recreational services, vocational training services, or services provided by agencies that finance care, provide transportation.

e This includes community health workers, health volunteers, and peer educators.

f Although countries may prioritize services according to the local situation, the range of services that adolescents require usually includes mental health, sexual and reproductive health, HIV, nutrition and physical activity, injuries and violence, substance misuse, and immunization. To inform countries' efforts in articulating national packages of adolescent health services, see World Health Organization recommended services and interventions for adolescents http://apps.who.int/adolescent/second-decade/section6/page1/universal-health-coverage.html.

g Standard operating procedures are desired where possible; these should be periodically updated.

h Services in the community may be provided by a wide range of both voluntary and paid health providers that work within and among the community and are often referred to as community health workers.

i Evidence-based management in line with guidelines and protocols is covered in Standard 4.

j The required competencies of staff should be clear in job descriptions.

k Competencies should encompass all areas of the package (e.g., mental health, sexual and reproductive health, violence prevention) and the entire range of services as delineated in Standard 3 (information, counseling, diagnosis, treatment and care).

l This includes right to information, privacy, confidentiality, nondiscrimination, nonjudgmental attitude, and respectful care.

m Effectiveness should be measured against evidence-based standards of care.

n This includes a comfortable seating area, available drinking water, educational materials in local language(s) that are attractive to adolescents, clean surroundings, waiting area, and toilets.

o These should address the following: (1) registration—information on the identity of the adolescent and the presenting issue are gathered in confidence; (2) consultation—confidentiality is maintained throughout the visit of the adolescent to the point of health service delivery (i.e., before, during and after a consultation); (3) record keeping—case records are kept in a secure place, accessible only to authorized personnel; and (4) disclosure of information—staff do not disclose any information given to or received from an adolescent to third parties such as family members, school teachers, or employers without the adolescent's consent (except where staff are obliged by legal requirements to report incidents such as sexual assaults, road traffic accidents or gunshot wounds to the relevant authorities).

p This includes the experience of care alongside *all dimensions* of quality of care as outlined in these standards (e.g., access to information, staff attitude, communication, guideline-driven care).

q This criterion can be measured by comparing the experience of care in groups of adolescents with various socioeconomic characteristics.

r For example, peer educators, counselors, and trainers.

s Other characteristics, such as schooling or marital status, are important to assess how equitable the service is, for example, whether in-school or out-of-school adolescents have same access to and use of services. However, in some contexts, adolescents may perceive that being asked, for example, about marital status is a barrier to the use of the service. Due consideration is required, therefore, so that data collection does not preclude adolescents' access to services.

t This includes the assessment of adolescents' experience of care (see Standard 8).

u This may include adolescents' perceived health care needs and adolescents' opinions on what services should be provided, as well as aspects of organization (e.g., working hours), provider-related aspects (e.g., strong preference for male or female provider), and other aspects.

v For each option, evidence-based information on advantages, disadvantages, and consequences should be provided; communication with the adolescent is in a language and format he/she can understand.

w For example, peer education.

## Box-1 Key questions for the peer-review process

1. Is the document clear and terms used consistent?
2. Do the eight standards and their criteria cover all important aspects of quality of health care services for adolescents? If not, what is missing?
3. Within each standard, are all the essential criteria listed? Conversely, are there criteria that are not essential?
4. Are there redundancies between criteria within each standard and between criteria across standards?

## Supplementary Data

Appendices A and B
