# Supplementary material for: Improving the Quality of Health Care Services for Adolescents, Globally: A Standards-Driven Approach
Source: J Adolesc Health. 2015 Sep;57(3):288–98. doi: 10.1016/j.jadohealth.2015.05.011 (PMC4540599; doi:10.1016/j.jadohealth.2015.05.011)
Supplement: Appendices A and B [file mmc1.doc]

## Appendix-A: Databases and key search terms

### Databases searched

Databases searched for published literature were Cochrane, MEDLINE, EMBASE, CINAHL (Cumulative Index to Nursing and Allied Health Literature), PubMed Clinical Queries, Database of Abstracts of Reviews of Effects (DARE), Health Services Research (HSR), PubMed Queries and Regional databases of the WHO - Global health library. In addition we hand-searched all 16 volumes of the Journal – ‘Quality and Safety in Health Care’ (previously Quality in Health Care) and the reference lists of the included studies. The grey literature searches were restricted to dissertations, reports and conference proceedings. The two recognized databases ‘Index to theses’ and ‘Proquest Dissertations & Theses’ were used. In addition, we also searched Google scholar and the advance online access notices of Cochrane Library.

### Search codes

### MEDLINE

adolescent[Title/Abstract] OR adolescence[Title/Abstract] OR "young people"[Title/Abstract] OR "youth friendly"[Title/Abstract] OR youth[Title/Abstract] OR teenage[Title/Abstract] OR teenager[Title/Abstract] OR teen[Title/Abstract] AND quality[Title/Abstract] AND care[Title/Abstract] OR healthcare[Title/Abstract] OR "health service"[Title/Abstract] AND ((Meta-Analysis[ptyp] OR systematic[sb]) AND ("2000/01/01"[PDAT] : "2013/12/31"[PDAT]) AND "humans"[MeSH Terms] AND systematic[sb])

AND

"delivery of health care"[MeSH Terms] OR "health services"[MeSH Terms] OR "adolescent"[MeSH Terms] AND ((Meta-Analysis[ptyp] OR systematic[sb]) AND ("2000/01/01"[PDAT] : "2013/12/31"[PDAT]) AND "humans"[MeSH Terms] AND systematic[sb])

### COCHRANE

(Title, abstract, keywords)

adolescent or adolescence or "young people" or "youth friendly" or youth or teenage or teenager or teen and quality and care or healthcare or "health service" or "performance assessment" or "quality assessment" or "quality improvement" or "performance improvement" or "quality assurance" or audit or monitoring or evaluation or supervision or feedback or mentoring or regulation or standard or accreditation:ti,ab,kw from 2000 to 2013, in Cochrane Reviews (Reviews only) (Word variations have been searched)

### EMBASE

1. adolescent OR adolescence OR "young people" OR “youth friendly” OR youth OR teenage OR teenager OR teen AND quality AND care OR healthcare OR “health service” OR “performance assessment” OR “quality assessment” OR “quality improvement” OR “performance improvement” OR “quality assurance” OR audit OR monitoring OR evaluation OR supervision OR feedback OR mentoring OR regulation OR standard OR accreditation {Including Related Terms}
2. limit 3 to (latest update and human and (meta analysis or "systematic review") and yr="2000 - 2013" and article and infant <to one year>)

### CINAHL

**Search terms -** adolescent OR adolescence OR young people OR youth friendly OR youth OR teenage OR teenager OR teen AND quality AND care OR healthcare OR health service OR performance assessment OR quality assessment OR quality improvement OR performance improvement OR quality assurance OR audit OR monitoring OR evaluation OR supervision OR feedback OR mentoring OR regulation OR standard OR accreditation

**Limiters -** Published Date from: 20000101-20130331; Exclude MEDLINE records; Publication Type: Meta Analysis, Meta Synthesis, Systematic Review

**Expanders -** Also search within the full text of the articles

**Search modes -** Find all my search terms

### WHO REGIONAL DATABASES

1. Adolescen$ AND quality AND care AND review
2. Youth$ AND quality AND care AND review
3. Teen$ AND quality AND care AND review
4. Adolescen$ AND quality AND healthcare AND review
5. Youth$ AND quality AND healthcare AND review
6. Teen$ AND quality AND healthcare AND review
7. Adolescen$ AND quality AND assessment AND review
8. Youth$ AND quality AND assessment AND review
9. Teen$ AND quality AND assessment AND review
10. Adolescen$ AND quality AND assurance AND review
11. Youth$ AND quality AND assurance AND review
12. Teen$ AND quality AND assurance AND review
13. Adolescen$ AND quality AND improvement AND review
14. Youth$ AND quality AND improvement AND review
15. Teen$ AND quality AND improvement AND review
16. Adolescen$ AND performance AND improvement AND review
17. Youth$ AND performance AND improvement AND review
18. Teen$ AND performance AND improvement AND review
19. Adolescen$ AND performance AND assessment AND review
20. Youth$ AND performance AND assessment AND review
21. Teen$ AND performance AND assessment AND review
22. Adolescen$ AND audit AND review
23. Youth$ AND audit AND review
24. Teen$ AND audit AND review
25. Adolescen$ AND monitor$ AND review
26. Youth$ AND monitor$ AND review
27. Teen$ AND monitor$ AND review
28. Adolescen$ AND evaluat$ AND review
29. Youth$ AND evaluat$ AND review
30. Teen$ AND evaluat$ AND review
31. Adolescen$ AND regulat$ AND review
32. Youth$ AND regulat$ AND review
33. Teen$ AND regulat$ AND review
34. Adolescen$ AND feedback AND review
35. Youth$ AND feedback AND review
36. Teen$ AND feedback AND review
37. Adolescen$ AND supervision AND review
38. Youth$ AND supervision AND review
39. Teen$ AND supervision AND review
40. Adolescen$ AND standard AND review
41. Youth$ AND standard AND review
42. Teen$ AND standard AND review
43. Adolescen$ AND mentor$ AND review
44. Youth$ AND mentor$ AND review
45. Teen$ AND mentor$ AND review
46. Adolescen$ AND accredit$ AND review
47. Youth$ AND accredit$ AND review
48. Teen$ AND accredit$ AND review

# Appendix-B: Lists of included and excluded studies

Full-texts included

| Sl. No. | Full citation |
| --- | --- |
| 1 | Beach MC, Gary TL, Price EG, Robinson K, Gozu A, Palacio A, et al. Improving health care quality for racial/ethnic minorities: a systematic review of the best evidence regarding provider and organization interventions. BMC Public Health. 2006;6:104. |
| 2 | Elster A, Jarosik J, VanGeest J, Fleming M. Racial and ethnic disparities in health care for adolescents: A systematic review of the literature. Archives of pediatrics & adolescent medicine. 2003;157(9):867-74. |
| 3 | Militello LK, Kelly SA, Melnyk BM. Systematic Review of Text-Messaging Interventions to Promote Healthy Behaviors in Pediatric and Adolescent Populations: Implications for Clinical Practice and Research. Worldviews on Evidence-Based Nursing. 2012;9(2):66-77. |
| 4 | Oringanje C, Meremikwu Martin M, Eko H, Esu E, Meremikwu A, Ehiri John E. Interventions for preventing unintended pregnancies among adolescents. Cochrane Database of Systematic Reviews. 2009(4). |
| 5 | Salema NE, Elliott RA, Glazebrook C. A systematic review of adherence-enhancing interventions in adolescents taking long-term medicines. The Journal of adolescent health : official publication of the Society for Adolescent Medicine. 2011;49(5):455-66. |
| 6 | Mason-Jones AJ, Crisp C, Momberg M, Koech J, Koker P, Mathews C. A systematic review of the role of school-based healthcare in adolescent sexual, reproductive, and mental health. Systematic Reviews. 2012(1). |
| 7 | Speizer IS, Magnani RJ, Colvin CE. The effectiveness of adolescent reproductive health interventions in developing countries: a review of the evidence Journal of Adolescent Health. 2003;33(5):324-48. |
| 8 | Dean AJ, Walters J, Hall A. A systematic review of interventions to enhance medication adherence in children and adolescents with chronic illness. Archives of Disease in Childhood. 2010;95(9):717-23. |
| 9 | Letourneau NL, Stewart MJ, Barnfather AK. Adolescent mothers: support needs, resources, and support-education interventions. Journal of Adolescent Health. 2004;35(6):509-25. |
| 10 | Stinson J, Wilson R, Gill N, Yamada J, Holt J. A systematic review of internet-based self-management interventions for youth with health conditions. Journal of Pediatric Psychology. 2009;35(5):495-510. |
| 11 | Ruiz-Mirazo E, Lopez-Yarto M, McDonald SD. Group prenatal care versus individual prenatal care: a systematic review and meta-analyses. Journal of obstetrics and gynaecology Canada. 2012;34(3):223-9. |
| 12 | Hall Moran V, Edwards J, Dykes F, Downe S. A systematic review of the nature of support for breast-feeding adolescent mothers. Midwifery. 2007;23(2):157-71. |
| 13 | Ambresin A-E, Bennett K, Patton GC, Sanci LA, Sawyer SM. Assessment of Youth-Friendly Health Care: A Systematic Review of Indicators Drawn From Young People's Perspectives. Journal of Adolescent Health. 2013;52(6):670-81. |

Full – texts excluded

| Sl. No. | Full citation | Reasons for excluding |
| --- | --- | --- |
| 1 | Hedberg VA, Klein JD, Andresen E. Health counseling in adolescent preventive visits: effectiveness, current practices, and quality measurement. Journal of Adolescent Health. 1998;23(6):344-53. | Not a systematic review |
| 2 | Allen J, Gamble J, Stapleton H, Kildea S. Does the way maternity care is provided affect maternal and neonatal outcomes for young women? A review of the research literature. Women and Birth. 2012;25(2):54-63. | Not a systematic review |
| 3 | Deenadayalan Y, Perraton L, Machotka Z, Kumar S. Day therapy programs for adolescents with mental health problems: a systematic review. Internet Journal of Allied Health Sciences & Practice. 2010;8(1):1-14. | Specific disease condition – exclusion criteria |
| 4 | Macdonald G, Bennett C, Dennis Jane A, Coren E, Patterson J, Astin M, et al. Home-based support for disadvantaged teenage mothers. Cochrane Database of Systematic Reviews. 2008(1). | Withdrawn by authors due to errors |
| 5 | Macdonald G, Turner W. Treatment Foster Care for improving outcomes in children and young people. Cochrane Database of Systematic Reviews. 2008(1). | Special disease condition – exclusion criteria |
| 6 | Martin S, Sutcliffe P, Griffiths F, Sturt J, Powell J, Adams A, et al. Effectiveness and impact of networked communication interventions in young people with mental health conditions: a systematic review. Patient Education and Counseling. 2011;85(2):e108-e19. | Specific disease condition – exclusion criteria |
| 7 | Mbuagbaw L, Ye C, Thabane L. Motivational interviewing for improving outcomes in youth living with HIV. Cochrane Database of Systematic Reviews. 2012(9). | Specific disease condition – exclusion criteria |
| 8 | Oude Luttikhuis H, Baur L, Jansen H, Shrewsbury Vanessa A, O'Malley C, Stolk Ronald P, et al. Interventions for treating obesity in children. Cochrane Database of Systematic Reviews. 2009(1). | Specific disease condition – exclusion criteria |
| 9 | Pippens RC, Hendricks-Ferguson VL, Peters-Herron A, Hufker K. Evidence-Based Analysis of the Incidence and Current Health Care Management for Preeclampsia Among Adolescent Mothers. JOGNN: Journal of Obstetric, Gynecologic & Neonatal Nursing. 2010;39:S106-7. | Not a systematic review |
| 10 | Shepherd Jonathan P, Frampton Geoff K, Harris P. Interventions for encouraging sexual behaviours intended to prevent cervical cancer. Cochrane Database of Systematic Reviews. 2011(4). | Behaviour change intervention for specific health conditions – exclusion criteria |
| 11 | Stein RE, Zitner LE, Jensen PS. Interventions for adolescent depression in primary care. Pediatrics. 2006(2):669-82. | Specific disease condition – exclusion criteria |
| 12 | Williams SB, O'Connor EA, Eder M, Whitlock EP. Screening for Child and Adolescent Depression in Primary Care Settings: A Systematic Evidence Review for the US Preventive Services Task Force. Pediatrics. 2009;123(4):e716-e35. | Specific disease condition – exclusion criteria |
| 13 | Rotter T, Kinsman L, James Erica L, Machotta A, Gothe H, Willis J, et al. Clinical pathways: effects on professional practice, patient outcomes, length of stay and hospital costs. Cochrane Database of Systematic Reviews. 2010(3). | Does not include adolescents relevant to the review |
| 14 | Rotter T, Kugler J, Koch R, Gothe H, Twork S, Oostrum JM, et al. A systematic review and meta-analysis of the effects of clinical pathways on length of stay, hospital costs and patient outcomes. BMC Health Services Research. 2008;8(1):265. | Data on children could not be separated from adults, also an older review of citation 13 |
| 15 | Szilagyi PG, Rand CM, McLaurin J, Tan L, Britto M, Francis A, et al. Delivering adolescent vaccinations in the medical home: a new era? Pediatrics. 2008;121 Suppl 1:S15-24. | Not a systematic review |
| 16 | Strunk JA. The effect of school-based health clinics on teenage pregnancy and parenting outcomes: an integrated literature review. Journal of School Nursing. 2008;24(1):13-20. | Not a systematic review |
| 17 | Tylee A, Haller DM, Graham T, Churchill R, Sanci LA. Youth-friendly primary-care services: how are we doing and what more needs to be done? The Lancet. 2007;369(9572):1565-73. | Not a systematic review |
| 18 | Campbell F, O'Neill Philip M, While A, McDonagh J. Interventions to improve transition of care for adolescents from paediatric services to adult services. Cochrane Database of Systematic Reviews. 2012(4). | Study protocol |
| 19 | Cardoza VJ, Documét PI, Fryer CS, Gold MA, Butler J. Sexual health behavior interventions for U.S. Latino adolescents: a systematic review of the literature. Journal of Pediatric and Adolescent Gynecology. 2012;25(2):136-49. | Mostly school-based interventions, a few studies have included interventions involving health facility and/or health provider, but findings could not be isolated for these |
| 20 | Fitzpatrick E, Walton-Moss B. Barriers to Emergency Contraception for Adolescents. Journal for Nurse Practitioners. 2011;7(4):282-6. | Not a systematic review |
| 21 | Jaskiewicz MG. An integrative review of the health care needs of female adolescents. Journal for Nurse Practitioners. 2009;5(4):274-83. | Not a systematic review |
| 22 | Donkoh C, Underhill K, Montgomery P. Independent living programmes for improving outcomes for young people leaving the care system. Cochrane Database of Systematic Reviews. 2006(3). | Not related to health system. The review is on “Public Care Homes” for children and adolescents |
| 23 | de Barros LP, Gropo LN, Petribú K, Colares V. Avaliação da qualidade de vida em adolescentes–revisão da literatura. J Bras Psiquiatr. 2008;57(3):212-7.  (Portuguese paper) | Not a systematic review |
| 24 | Henriques BD, Rocha RL, Madeira AMF. Saúde do adolescente: o significado do atendimento para os profissionais da atenção primária do município de Viçosa, MG. REVISTA MÉDICA DE MINAS GERAIS-RMMG. 2010;20(3). | Not a systematic review |
